# The Use of Permanganate of Potash

**Published:** 1866-07

**Authors:** 


THE USE OF PERMANGANATE OF POTASH.

Dr. B. F. Craig read a paper on the use of permanganate of potash for the purification of water, especially during the prevalence of epidemic cholera.

The points of the paper were presented by Drs. C. A. Lee and E. R. Squibb, namely, that the disinfecting agent in the use of the permanganate of potassa and allied substances—was ozone. According to the remarks of Dr. Squibb, the power of disinfecting substances generally, excepting some, such as charcoal, which are absorbents, can be measured in a certain degree by the facility with which they give out or abstract oxygen. The paper under discussion was defective, in that the author assumed that the organic matter present in nature is the cause of cholera; and also that he took no note of the deposit of carbonate of potassa that would take place even to a mischievous degree in the use of the remedy. Yet, upon the whole, the paper should be regarded as a contribution to the important subject of disinfectants. It was, on motion, voted to commend it to the Committee on Publication.

